# The effects of transurethral resection and cystoprostatectomy on dissemination of epithelial cells in the circulation of patients with bladder cancer

**DOI:** 10.1038/sj.bjc.6690771

**Published:** 1999-11

**Authors:** F Desgrandchamps, M Teren, L Dal Cortivo, J-P Marolleau, P Bertheau, J-M Villette, A Cortesse, P Teillac, A Le Duc, F C Hamdy

**Affiliations:** Departments of Urology, ETS-AP-HP, Hormonal Biochemistry andPathology, Hôpital Saint-Louis, Paris, France; Section of Urology, University of Sheffield Medical School, Royal Hallamshire Hospital, I Floor, Glossop Road, Sheffield S10 2JF, UK

**Keywords:** bladder cancer, circulating epithelial cells, transurethral resection, cystoprostatectomy, metastasis

## Abstract

This study was undertaken to evaluate the risk of haematogenous dissemination of epithelial cells induced by endoscopic resection and/or cystoprostatectomy for transitional cell carcinoma of the bladder. Thirty-three patients were studied. Thirty-one had different stages and grades of bladder cancer and two patients had benign bladder conditions. Twenty-five cancer patients required transurethral resection of their bladder tumour. Of those, 20 had superficial disease (pTaG1–G2: *n* = 19; pT1G2: *n* = 1) and five had muscle invasive tumours (pT2G3: *n* = 2; pT3aG3: *n* = 1; pT4G3: *n* = 2). Five patients underwent radical cystoprostatectomy for muscle invasive cancers (pT2G3: *n* = 3; pT3bG3: *n* = 1; pT4G3: *n* = 1) and one man received chemotherapy for metastatic disease. Venous blood (10 ml) was obtained from the antecubital fossa in each patient, before and 1–2 h after completion of surgery, and prior to treatment in the metastatic patient. An indirect immunocytochemical technique was used to detect circulating epithelial cells after centrifugation on Ficoll gradient and fixation of mononuclear cells on slides, using a monoclonal antibody directed against three cytokeratins: CK8, CK18 and CK19. Circulating epithelial cells were detected only in the patient with metastatic disease. None of the other patients had evidence of epithelial circulating cells before or after surgery. The results suggest that irrespective of disease stage and grade, neither endoscopic nor open bladder surgery leads to detectable dissemination of urothelial cells in the peripheral circulation. These procedures are therefore unlikely to increase the risk of progression and metastasis in transitional cell carcinoma of the bladder. © 1999 Cancer Research Campaign


*British Journal of Cancer* (1999) **81**(5), 832–834
© 1999 Cancer Research Campaign
Article no. bjoc.1999.0771

### The effects of transurethral resection and cystoprostatectomy on dissemination of epithelial cells in the circulation of patients with bladder cancer

**F Desgrandchamps[1], M Teren[1], L Dal Cortivo[2], J-P Marolleau[2], P Bertheau[4], J-M Villette[3], A Cortesse[1], P Teillac[1], A Le Duc[1] and FC Hamdy[5]**

Departments of [1]Urology, [2]ETS-AP-HP, [3]Hormonal Biochemistry and [4]Pathology, Hôpital Saint-Louis, Paris, France; [5]Section of Urology, University of Sheffield Medical School, Royal Hallamshire Hospital, I Floor, Glossop Road, Sheffield S10 2JF, UK

**Summary** This study was undertaken to evaluate the risk of haematogenous dissemination of epithelial cells induced by endoscopic resection and/or cystoprostatectomy for transitional cell carcinoma of the bladder. Thirty-three patients were studied. Thirty-one had different stages and grades of bladder cancer and two patients had benign bladder conditions. Twenty-five cancer patients required transurethral resection of their bladder tumour. Of those, 20 had superficial disease (pTaG1–G2: $n$ = 19; pT1G2: $n$ = 1) and five had muscle invasive tumours (pT2G3: $n$ = 2; pT3aG3: $n$ = 1; pT4G3: $n$ = 2). Five patients underwent radical cystoprostatectomy for muscle invasive cancers (pT2G3: $n$ = 3; pT3bG3: $n$ = 1; pT4G3: $n$ = 1) and one man received chemotherapy for metastatic disease. Venous blood (10 ml) was obtained from the antecubital fossa in each patient, before and 1–2 h after completion of surgery, and prior to treatment in the metastatic patient. An indirect immunocytochemical technique was used to detect circulating epithelial cells after centrifugation on Ficoll gradient and fixation of mononuclear cells on slides, using a monoclonal antibody directed against three cytokeratins: CK8, CK18 and CK19. Circulating epithelial cells were detected only in the patient with metastatic disease. None of the other patients had evidence of epithelial circulating cells before or after surgery. The results suggest that irrespective of disease stage and grade, neither endoscopic nor open bladder surgery leads to detectable dissemination of urothelial cells in the peripheral circulation. These procedures are therefore unlikely to increase the risk of progression and metastasis in transitional cell carcinoma of the bladder. © 1999 Cancer Research Campaign

**Keywords:** bladder cancer; circulating epithelial cells; transurethral resection; cystoprostatectomy; metastasis

Transurethral resection of exophytic bladder tumours (TURT) is the first step in the management of bladder cancer. The procedure confirms the diagnosis and provides important prognostic information, including grading and staging of the disease. During endoscopy, the tumour and underlying muscle are resected. This procedure, performed under continuous irrigation, can lead to passage of the irrigating fluid, accompanied by benign and/or malignant epithelial cell suspensions, into the peripheral circulation, facilitated by inevitable venous breaches during resection, and by transient elevations of the intravesical pressure during resection (Hahn, 1995). Theoretically, this may contribute to disease progression, invasion and metastases as has been suggested in other malignancies, including breast and prostate cancer during open surgical excision which has been shown to induce passage of normal and malignant epithelial cells into the peripheral circulation (Eschwège et al, 1995; Choy and McCulloch, 1996; Oeflein et al, 1996). To our knowledge, however, haematogenous dissemination of epithelial cells during endoscopic resection of bladder cancer or during cystoprostatectomy has not been studied previously.

The aim of our study was to evaluate prospectively the risk of haematogenous dissemination of epithelial cells following transurethral resection and cystoprostatectomy for bladder cancer.

*Received 17 September 1998*
*Revised 12 February 1999*
*Accepted 8 June 1999*

*Correspondence to*: FC Hamdy

### PATIENTS, MATERIALS AND METHODS

### Patients

Thirty-three patients were studied. Two were thought to have malignant disease in the bladder, but were found to have non-specific inflammation ($n$ = 1) and schistosomiasis ($n$ = 1) with no evidence of malignancy. Thirty-one consecutive patients had different stages and grades of transitional cell carcinoma of the bladder, 25 of whom required transurethral resection of their bladder tumour. Of those, 20 had superficial disease (pTaG1–G2: $n$ = 19; pT1G2: $n$ = 1), all tumours being single or multifocal with a total fresh weight less than 5 g, and no post-operative haemorrhagic complications; and five had muscle invasive tumours (pT2G3: $n$ = 2; pT3aG3: $n$ = 1; pT4G3: $n$ = 2). Five of the remaining cancer patients underwent radical cystoprostatectomy for muscle invasive cancers (pT2G3: $n$ = 3; pT3bG3: $n$ = 1; pT4G3: $n$ = 1). The final patient who was analysed received a radical cystoprostatectomy 6 months earlier for a pT4G3 tumour, and developed pelvic recurrence and skeletal metastases. He was treated with systemic chemotherapy.

### Sample collection and preparation

Venous blood samples (10 ml) were obtained by venepuncture from the antecubital fossa in every patient before surgery, and between 1 and 2 h after completion of the procedure. In the patient with known metastatic disease, blood samples were obtained prior to administration of chemotherapy.

The samples were diluted with phosphate-buffered saline (PBS) and resuspended in Dulbecco's modified Eagle's medium (Gibco, UK). Mononuclear cells were isolated by Ficoll-hypaque (density 1.011), washed twice in PBS and resuspended at $1 \times 10^6$ mononuclear cells $ml^{-1}$ in PBS. Cells were spun at $0.5 \times 10^6$ cells per slide in 0.5 ml. Cytospin slides were prepared using a centrifuge, air-dried for 12–24 h and fixed in acetone for 10 min before freezing. Five cytospins per sample were prepared, allowing the analysis of a total of $2.5 \times 10^6$ mononuclear cells for each sample.

### Immunocytochemistry

Cytospin slides were incubated with a monoclonal antibody which detects a common epitope of a variety of cytokeratin components, including CK8, CK18 and CK19 (Micromet®, Martinsried, Germany), expressed by bladder epithelial cells (Moll et al, 1982). After incubation, the antibody reaction was developed using the indirect immunoenzyme alkaline phosphatase anti-alkaline phosphatase (APAAP) technique. Red cytoplasmic staining of positive cells was assessed by light microscopy. Slides were evaluated by two independent observers (LDC and PB) in a double-blind fashion.

### Validation studies

In order to test tumour cell losses during global processing, T24 (a human urothelial carcinoma cell line derived from the American Type Tissue Collection, T24 (Bubenik et al, 1973) (reference ATCC HTB-1)) were spiked in whole heparinized blood samples, to a density of 1 cell per 100 mononuclear cells. T24 cell recovery was evaluated after Ficoll gradient extraction and immunocytochemistry. Sensitivity of the technique was evaluated by spiking T24 cells at different concentrations in mononuclear cells obtained from healthy donors, ranging from 1 tumour cell per $10^5$ mononuclear cells to 1 tumour cell per $10^6$ mononuclear cells.

### Immunocytochemical internal control

T24 cells were mixed with heparinized blood from a healthy donor to a density of 1 cell per 100 mononuclear cells. Cytospins were prepared and labelled with the anti-pancytokeratine 8–19 monoclonal antibody.

### RESULTS

### Circulating epithelial cells

In all 33 patients who were studied, one blood sample showed evidence of epithelial cells. This sample was obtained from the patient with known metastatic disease who had not undergone surgery immediately prior to blood sampling. All the other patients had no evidence of any epithelial cells in the circulation.

### Validation studies

Mean tumour cell recovery was 85% (mean of three experiments). It was possible to detect up to 1 tumour cell per $5 \times 10^5$ mononuclear cells.

### Immunocytochemical internal control

The T24 cell line was uniformly recognized after culture by the anti-pancytokeratin 8–18 and 19 monoclonal antibody. Labelling of the cytospins obtained from this culture constituted the immunocytochemical positive control for all patient blood samples tested.

### DISCUSSION

To our knowledge, the detection of circulating epithelial cells after urinary bladder surgery has not been studied previously. By using an immunocytochemical detection technique, we showed that neither endoscopic resection nor open surgery induced any significant passage of epithelial cells into the bloodstream. Immunocytochemical techniques based on the use of monoclonal antibodies directed against cytokeratins have been used for a long time to demonstrate circulating epithelial cells. Normal and neoplastic bladder epithelial cells are known to express cytokeratins CK8, CK18 and CK19 (Moll et al, 1982; Carbin et al, 1987). Soluble cytokeratin fragments, released by tumour cell necrosis, can be detected in the blood or urine of patients with bladder cancer. CYFRA 21-1 detects soluble fragments of CK19 (Senga et al, 1996; Stieber et al, 1996), and tissue polypeptide antigen (TPA) detects soluble fragments of CK8, CK18 and CK19 (Maulard et al, 1994; Sundstrom and Stigbrand, 1994). Serum CYFRA 21-1 and TPA levels do not increase after endoscopic resection of bladder tumours (Maulard et al, 1994; Senga et al, 1996), suggesting the absence of any significant vascular passage of cell debris during resection. These data support the results of our study.

Immunocytochemistry has demonstrated the presence of epithelial cells in the efferent blood of kidney (Glaves et al, 1988) and colonic tumours (Leather et al, 1993), and showed that surgical resection of breast tumours could induce the passage of epithelial cells into the distal venous blood circulation (Choy and McCulloch, 1996). This technique can demonstrate circulating epithelial cells beyond a density of 10 epithelial cells per ml of blood. Like reverse transcription polymerase chain reaction (RT-PCR) amplification, immunocytochemistry is able to detect in vitro 1 in $10^6$ mononuclear cells (Wood et al, 1994). Although RT-PCR detection is a more sensitive technique than immunocytochemistry at this low concentration (Wood et al, 1994), no ideal RT-PCR amplification target is available for bladder epithelial cells in vivo. RT-PCR amplification of cytokeratin mRNA can induce a high risk of false-positives with CK8, CK18 and CK19 which, depending on the primers used, can be as high as 88% with CK8 and 40% with CK19 (Burchill et al, 1995; Zippelius et al, 1997). Furthermore, RT-PCR and immunohistochemistry were shown to have an equivalent sensitivity for demonstration of circulating bone marrow aspirate epithelial cells based on cytokeratin 18 expression (Zippelius et al, 1997).

Uroplakin gene (Up Ib and UP III) expression has been demonstrated recently by RT-PCR in the peripheral blood of patients with metastatic bladder cancer (Yuasa et al, 1998). It is likely that these genes will be useful and specific for urothelial cell detection in peripheral blood in the near future, but at present immunocytochemistry remains the reference technique for demonstration of non-prostatic circulating epithelial cells.

For practical reasons, blood samples were obtained from the antecubital fossa in our patients between 1 and 2 h after completion of endoscopic resection. Passage of circulating epithelial cells released from a pelvic organ via the pulmonary circulation does not prevent their detection in peripheral venous blood drawn from the antecubital fossa. Moreover, when epithelial cells are released from a tumour, they can be demonstrated in circulating blood for up to 3 days after surgical handling of this tumour (Eschwège et al,

*© 1999 Cancer Research Campaign*

1995; Oefelein et al, 1996). It is therefore unlikely for our protocol to have reduced the sensitivity of the detection technique we used. Our methods were validated further by the control experiments, and the fact that the only positive patient had known metastatic bladder cancer.

Despite the relatively small number of patients analysed, various stages of the disease were investigated. Although the biological behaviour of superficial bladder cancer differs from invasive disease, some non-invasive cancers do progress and metastasize, hence the importance of including this group of patients in our study to assess the potential contribution of surgery in the initiation of disease progression. Using the detection technique described, the results of our study suggest that irrespective of disease stage, neither TURT nor cystoprostatectomy led to significant dissemination of urothelial cells in the peripheral blood. It is therefore unlikely that these procedures increase the risk of distant metastases in patients with transitional cell carcinoma of the bladder. Further studies comparing different epithelial cell detection techniques in peripheral blood of patients with bladder cancer are warranted to substantiate our findings.

### REFERENCES

Bubenik J, Baresova M, Viklicky V, Jakoubrova J, Sainerova H, Donner J (1973) Established cell line of urinary bladder carcinoma (T24) containing tumor-specific antigen. *Int J Cancer* **11**: 765–773

Burchill SA, Bradbury MF, Pittman K, Southgate J, Smith B, Selby P (1995) Detection of epithelial cancer cells in peripheral blood by reverse transcriptase-polymerase chain reaction. *Br J Cancer* **71**: 278–281

Carbin BE, Collins VP, Ekman P (1987) Tissue polypeptide antigen (TPA), some cytokeratins and epithelial membrane antigen (EMA) in normal, inflamed and malignant urothelium. *Urol Res* **15**: 191–194

Choy A, McCulloch P (1996) Induction of tumor cell shedding into effluent venous blood breast cancer surgery. *Br J Cancer* **73**: 79–82

Eschwège P, Dumas F, Blanchet P, Le Maire V, Benoit G, Jardin A, Lacour B, Loric S (1995) Haematogenous dissemination of prostatic epithelial cells during radical prostatectomy. *Lancet* **346**: 1528–1530

Glaves D, Huben RP, Weiss L (1988) Haematogenous dissemination of cells from human renal adenocarcinomas. *Br J Cancer* **57**: 32–35

Hahn RG (1995) Transurethral resection syndrome after transurethral resection of bladder tumours. *Can J Anaesth* **42**: 69–72

Leather AJM, Gallegos NC, Kocjan G, Savage F, Smales CS, Hu W, Boulos PB, Northover JM and Phillips RK (1993) Detection and enumeration of circulating tumour cells in colorectal cancer. *Br J Cancer* **80**: 777–780

Maulard C, Toubert ME, Chretien Y, Delanian S, Dufour B and Housset M (1994) Serum tissue polypeptide antigen (S-TPA) in bladder cancer as tumor marker. A prospective study. *Cancer* **73**: 394–398

Moll R, Franke WW, Schiller DL, Geiger B and Krepler R (1982) The catalog of human cytokeratins: patterns of expression in normal epithelia, tumors and cultured cells. *Cell* **31**: 11–24

Oefelein MG, Kaul K, Herz B, Blum MD, Holland JM, Keeler TC, Cook WA and Ignatoff JM (1996) Molecular detection of prostate epithelial cells from the surgical field and peripheral circulation during radical prostatectomy. *J Urol* **155**: 238–242

Senga Y, Kimura G, Hattori T and Yoshida K (1996) Clinical evaluation of soluble cytokeratin 19 fragments (CYFRA 21-1) in serum and urine of patients with bladder cancer. *Urology* **48**: 703–710

Stieber P, Schmeller N, Schambeck C, Hofmann K, Reiter W, Hasholzner U and Fateh-Moghadam A (1996) Clinical relevance of CYFRA 21-1, TPA-IRMA and TPA-LIA-mat in urinary bladder cancer. *Anticancer Res* **16**: 3793–3798

Sundstrom BE and Stigbrand TI (1994) Cytokeratins and tissue polypeptide antigen. *Int J Biol Markers* **9**: 102–108

Wood DP, Banks ER, Humphreys S and Rangnekar VM (1994) Sensitivity of immunohistochemistry and polymerase chain reaction in detecting prostate cancer cells in bone marrow. *J Histochem Cytochem* **42**: 505–511

Yuasa T, Yoshiki T, Tanaka T, Kim CJ, Isono T and Okada Y (1998) Expression of uroplakin Ib and uroplakin III genes in tissues and peripheral blood of patients with transitional cell carcinoma. *Jpn J Cancer Res* **89**: 879–882

Zippelius A, Kufer G, Köllermann MW, Oberneder R, Schlimok G, Riethmüller G and Pantel K (1997) Limitations of reverse-transcriptase polymerase chain reaction analyses for detection of micrometastatic epithelial cancer cells in bone marrow. *J Clin Oncol* **15**: 2701–2708

© 1999 Cancer Research Campaign
